# Supplementary material for: A set of multi-entry identification keys to African frugivorous flies (Diptera, Tephritidae)
Source: Zookeys. 2014 Jul 24;(428):97–108. doi: 10.3897/zookeys.428.7366 (PMC4143993; doi:10.3897/zookeys.428.7366)
Supplement: Supplementary material 6 — Key to Ceratitis [file zookeys-428-097-s006.zip › SF6_ZooKeys_key to Ceratitis/key/SF6_ZooKeys_key to Ceratitis/Media/Html/Ceratitis morstatti .htm]

Ceratitis (Ceratalaspis) morstatti Bezzi


***Ceratitis*** **(*Pterandrus*) *morstatti*
Bezzi**

*Ceratitis morstatti* Bezzi, 1912: 12.

Body
length: 5.13 (4.50-5.70) mm; wing length: 5.34 (5.00-5.80) mm.

Male

Head: Antenna yellow-orange (yellow according to original
description). First flagellomere twice as long as pedicel. Arista with medium
long rays. Frons convex, yellow (dark red, with gray basal stripes according to
orginal description); with short scattered setulae distinctly darker than
frons. Genal seta black; genal setulae black, well developed.

Thorax: Postpronotal
lobe white, unspotted. Scutal pattern, ground color pale gray (occasionally
darker gray) with silvery shine; with very light streaks and darker markings
but without distinct spots except prescutellar yellow separate markings.
Scapular setae dark. Scutellum grayish, basally without spots, apically with
three separate black spots extending to halfway scutellar disc. Anepisternum on
ventral margin yellowish brown; setulae white, along ventral margin black.

Legs:
Yellow; setation typical for subgenus, mainly dark; without feathering.

Wing:
Bands yellow and brown. Interruption between marginal and discal bands
near vein R_1_ complete; cubital band free (only occasionally weakly
connected); medial band absent; crossvein R-M opposite middle of discal cell.
Apex of vein R_1_ distal to level of crossvein R-M. Crossvein DM-Cu
variable.

Abdomen:
Mostly gray. Tergites 2 and 4 with pale gray band occupying most if not whole
of tergite. Tergite 3 dark brown posteriorly. Tergite 5 with yellowish brown
and brown patches. Male terminalia not dissected.

Female

As
male except for the following characters. Oviscape as long as preabdomen.
Aculeus at least eight times longer than wide; tip with distinct apical
indentation and lateral margin slightly sinuous.

(Description
after De Meyer & Freidberg, 2006)
